# Supplementary material for: Causal Relationship between Aging and Anorexia Nervosa: A White-Matter-Microstructure-Mediated Mendelian Randomization Analysis
Source: Biomedicines. 2024 Aug 16;12(8):1874. doi: 10.3390/biomedicines12081874 (PMC11351342; doi:10.3390/biomedicines12081874)
Supplement: Supplementary file 1 [file biomedicines-12-01874-s001.zip › Supplementary Figure/Figure S8/Leaveoneout plot of MR analysis for TL to ubm-b-1979.pdf]

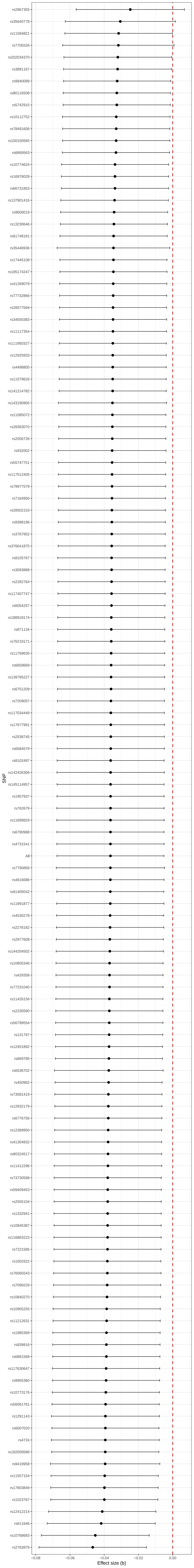
rs2967355
rs35640778
rs11584821
rs7705526
rs202034370
rs3891167
rs9940099
rs80116508
rs5742915
rs10112752
rs78491606
rs150150565
rs6669563
rs10774624
rs16978028
rs66731853
rs137901416
rs9600019
rs13230646
rs61748181
rs35446936
rs17445108
rs185174247
rs41269079
rs77732866
rs28577594
rs34550383
rs11117354
rs111950327
rs12925933
rs4498805
rs11579626
rs141214782
rs143190905
rs11085072
rs28363070
rs2056726
rs932002
rs55747751
rs117512405
rs79977579
rs7164950
rs28502153
rs9398196
rs3767952
rs376641875
rs8105767
rs3093888
rs2282764
rs117407747
rs6054257
rs188918174
rs871134
rs76219171
rs11769630
rs6659669
rs139795227
rs6751209
rs7209057
rs117034449
rs17677991
rs2538745
rs6584579
rs8102497
rs142426306
rs145114957
rs1957937
rs762679
rs11699829
rs6790988
rs4731541
All
rs7790856
rs4616688
rs61405042
rs11991877
rs4530278
rs2276182
rs2977608
rs144204502
rs10805346
rs429358
rs77231040
rs11426156
rs2230590
rs56799554
rs131797
rs12451892
rs869785
rs6536702
rs450962
rs73581419
rs12932179
rs6776756
rs12369950
rs41304832
rs80324517
rs11412296
rs73730598
rs59409453
rs2555104
rs1332941
rs10845387
rs116863223
rs7221585
rs1003322
rs76065543
rs7099229
rs10840270
rs10905255
rs11212631
rs1985369
rs939916
rs6881568
rs117630647
rs9955360
rs10773176
rs56061761
rs1291143
rs6007020
rs4724
rs182059586
rs9419958
rs11557154
rs17803849
rs1023767
rs12412214
rs611646
rs10768683
rs2763979
SNP
-0.08
-0.06
-0.04
-0.02
0.00
Effect size (b)
